# Reduction of estimated fluid volumes following initiation of empagliflozin in patients with type 2 diabetes and cardiovascular disease: a secondary analysis of the placebo-controlled, randomized EMBLEM trial

**DOI:** 10.1186/s12933-021-01295-6

**Published:** 2021-06-28

**Authors:** Atsushi Tanaka, Michio Shimabukuro, Hiroki Teragawa, Yosuke Okada, Toshinari Takamura, Isao Taguchi, Shigeru Toyoda, Hirofumi Tomiyama, Shinichiro Ueda, Yukihito Higashi, Koichi Node, the EMBLEM Investigators

**Affiliations:** 1 Department of Cardiovascular Medicine, Saga University, 5-5-1 Nabeshima, Saga, 849-8501 Japan; 2 Department of Diabetes, Endocrinology, and Metabolism, Fukushima Medical University, Fukushima, Japan; 3 Department of Cardiovascular Medicine, JR Hiroshima Hospital, Hiroshima, Japan; 4 First Department of Internal Medicine, School of Medicine, University of Occupational and Environmental Health, Japan, Kitakyusyu, Japan; 5 Department of Endocrinology and Metabolism, Kanazawa University Graduate School of Medical Sciences, Kanazawa, Japan; 6 Department of Cardiology, Dokkyo Medical University Saitama Medical Center, Koshigaya, Japan; 7 Department of Cardiovascular Medicine, Dokkyo Medical University School of Medicine, Mibu, Japan; 8 Department of Cardiology, Tokyo Medical University, Tokyo, Japan; 9 Department of Clinical Pharmacology and Therapeutics, University of the Ryukyus, Nishihara, Japan; 10 Department of Cardiovascular Regeneration and Medicine, Research Institute for Radiation Biology and Medicine, Hiroshima University, Hiroshima, Japan

**Keywords:** Fluid volume, Empagliflozin, Heart failure, Type 2 diabetes, Cardiovascular disease, N-terminal pro-brain natriuretic peptide

## Abstract

**Backgrounds/Aim:**

Sodium glucose co-transporter 2 inhibitors promote osmotic/natriuretic diuresis and reduce excess fluid volume, and this improves cardiovascular outcomes, including hospitalization for heart failure. We sought to assess the effect of empagliflozin on estimated fluid volumes in patients with type 2 diabetes and cardiovascular disease (CVD).

**Methods:**

The study was a *post-hoc* analysis of the EMBLEM trial (UMIN000024502), an investigator-initiated, multi-center, placebo-controlled, double-blinded, randomized-controlled trial designed primarily to evaluate the effect of 24 weeks of empagliflozin treatment on vascular endothelial function in patients with type 2 diabetes and established CVD. The analysis compared serial changes between empagliflozin (10 mg once daily, n = 52) and placebo (n = 53) in estimated plasma volume (ePV), calculated by the Straus formula and estimated the extracellular volume (eEV), determined by the body surface area, measured at baseline and 4, 12, and 24 weeks after initiation of treatment. Correlations were examined between the changes from baseline to week 24 in each estimated fluid volume parameter and several clinical variables of interest, including N-terminal pro-brain natriuretic peptide (NT-proBNP) concentration.

**Results:**

In an analysis using mixed-effects models for repeated measures, relative to placebo empagliflozin reduced ePV by − 2.23% (95% CI − 5.72 to 1.25) at week 4, − 8.07% (− 12.76 to − 3.37) at week 12, and − 5.60% (− 9.87 to − 1.32) at week 24; eEV by − 70.3 mL (95% CI − 136.8 to − 3.8) at week 4, − 135.9 mL (− 209.6 to − 62.3) at week 12, and − 144.4 mL (− 226.3 to − 62.4) at week 24. The effect of empagliflozin on these parameters was mostly consistent across various patient clinical characteristics. The change in log-transformed NT-proBNP was positively correlated with change in ePV (r = 0.351, p = 0.015), but not with change in eEV.

**Conclusions:**

Our data demonstrated that initiation of empagliflozin treatment substantially reduced estimated fluid volume parameters in patients with type 2 diabetes and CVD, and that this effect was maintained for 24 weeks. Given the early beneficial effect of empagliflozin on cardiovascular outcomes seen in similar patient populations, our findings provide an important insight into the key mechanisms underlying the clinical benefit of the drug.

*Trial registration* University Medical Information Network Clinical Trial Registry, number 000024502

**Supplementary Information:**

The online version contains supplementary material available at 10.1186/s12933-021-01295-6.

## Introduction

Previous cardiovascular outcome trials (CVOTs) have demonstrated that sodium glucose co-transporter 2 (SGLT2) inhibitors improve cardiovascular and renal outcomes, including hospitalization for heart failure (HHF), in patients with type 2 diabetes and a high risk of cardiovascular events [1]. Furthermore, recent CVOTs have shown that SGLT2 inhibitors reduce the risk of HHF and cardiovascular death in patients with established heart failure (HF) and reduced ejection fraction, regardless of their diabetic status [2], and even in patients who were admitted due to worsening HF [3]. Thus, SGLT2 inhibitors have beneficial effects on cardiovascular outcomes beyond their glucose-lowering action [4], and are now recommended to improve the prognosis of patients with type 2 diabetes and associated risk factors [5, 6]. However, the precise mechanisms of these benefits on cardiovascular disease and their clinical predictors following initiation of SGLT2 inhibitors are not fully understood [7, 8].

SGLT2 inhibitors promote osmotic/natriuretic diuresis and reduce excess plasma and interstitial volumes without affecting effective intravascular circulating volumes [9, 10]. Accordingly, this action of SGLT2 inhibitors appears to contribute substantially to the risk reduction in HHF [11]. In this context, a mediation analysis of data from the EMPA-REG OUTCOME (Empagliflozin Cardiovascular Outcome Event Trial in Type 2 Diabetes Mellitus Patients-Removing Excess Glucose) trial showed that increases in hematocrit and hemoglobin, indicative of the hemodynamic effect of SGLT2 inhibitors, were the strongest predictors of the reduction in the risk of cardiovascular death [12]. A similar effect was observed in another mediation analysis of data from the CANVAS (Canagliflozin Cardiovascular Assessment Study) Program [13]. Given that the effects of SGLT2 inhibitors on erythropoietic and volume status seem largely to mediate their cardiovascular benefits, some recent clinical studies showed that treatment with SGLT2 inhibitors reduced estimated fluid volume parameters in patients with type 2 diabetes [14] or HF with reduced ejection fraction [15].

In the EMBLEM (Effect of Empagliflozin on Endothelial Function in Cardiovascular High Risk Diabetes Mellitus: Multi-Center Placebo-Controlled Double-Blind Randomized) trial, 24 weeks of empagliflozin treatment did not affect endothelial function in patients with type 2 diabetes and established cardiovascular disease (**CVD**) [16]. Meanwhile, the erythropoietic parameters increased, and a parameter of estimated plasma volume (**ePV**) decreased 24 weeks after initiation of empagliflozin, relative to placebo [17]. To extend this initial observation regarding the plasma volume effect of empagliflozin, in the present *post-hoc* analysis of the EMBLEM trial we sought to further examine the serial changes in estimated fluid volume and their correlations with other clinical variables.

## Methods

### Study design and participants

The study was a *post-hoc* analysis of the EMBLEM trial (UMIN000024502), an investigator-initiated, multi-center, placebo-controlled, double-blinded, randomized-controlled trial undertaken in 16 centers in Japan. The EMBLEM trial was primarily designed to evaluate the effect of 24 weeks of empagliflozin treatment on vascular endothelial function as assessed by the reactive hyperemia index (RHI) in patients with type 2 diabetes and CVD. The details of the design, inclusion/exclusion criteria, and main results have been reported [16–18]. In brief, adults with type 2 diabetes, an HbA1c between 6.0 and 10.0%, and a history of at least one established CVD event (coronary artery disease, stroke, peripheral artery disease, the presence of known coronary artery stenosis (≥ 50%), or HF except for New York Heart Association classification IV) were included. Key exclusion criteria were a history of CVD, cerebrovascular disease, or coronary revascularization within 3 months before consent. Eligible patients were randomly assigned into either 24 weeks of treatment with empagliflozin (10 mg once daily) or placebo, using the web-based minimization dynamic allocation method, balancing for HbA1c (< 7.0 or ≥ 7.0%), age (< 65 or ≥ 65 years), systolic blood pressure (< 140 or ≥ 140 mmHg), and current smoking habit (smoker of nonsmoker) at the time of screening. Post-randomization follow-up visits were scheduled at weeks 4, 12, and 24.

The trial was approved by the institutional review boards of the individual sites. All participants received a detailed explanation of the trial and provided written informed consent.

### Fluid volume estimation

Estimations of fluid volumes were performed at baseline and at weeks 4, 12, and 24 after randomization using the following formulas and clinical variables. The ePV at baseline was calculated by the Kaplan-Hakim formula [19], and the percentage change in ePV by the Strauss formula [14, 15].

$$\text{Kaplan-Hakim formula: (1}-\mathrm{hematocrit}\text{)}\times (\mathrm{a}+[\mathrm{b}\times \mathrm{body weight }(\mathrm{kg})])*$$

* where a = 1530 in men and 864 in women, and b = 41 in men and 47.9 in women

$$\text{Strauss formula: 100}\, \times \frac{\text{hemoglobin }(\text{at baseline})}{\text{hemoglobin }(\text{at visit})}\times \frac{1-\text{hematocrit }(\text{at visit})}{1-\text{hematocrit }(\text{at baseline})}\,-\,100$$

The extracellular volume (**eEV**) was estimated by the following formula [15]:

$$\text{8116.6}\times [0.007184 \times \mathrm{ height }{\left(\mathrm{cm}\right)}^{0.725}\times \mathrm{weight }{\left(\mathrm{kg}\right)}^{0.425}]-\text{28.2}$$

### Study endpoint

The main endpoints in this *post-hoc* analysis were the between-group differences in the percentage change in ePV and the change in eEV from baseline to weeks 4, 12, and 24. In the empagliflozin arm, the correlations between the changes from baseline to week 24 in each estimated fluid volume and clinical variables of interest were also examined.

### Statistical analysis

Baseline characteristics are summarized as numbers (percentages) for categorical variables, and as means (with standard deviation) or medians (with interquartile range) for continuous variables, depending on the variable’s frequency distribution. Mean changes from baseline in ePV and eEV and their 95% confidence intervals (CI) were estimated by a longitudinal mixed-effects model for repeated measures (MMRM). The effects of empagliflozin vs. placebo on ePV and eEV at weeks 4, 12, and 24 were also assessed in several subgroups according to age, sex, body mass index (BMI), history of HF, diuretic use, and corresponding value at baseline. The proportional change from baseline to week 24 in the N-terminal pro-brain natriuretic peptide (NT-proBNP) concentration was calculated based on its logarithmic scale in the empagliflozin arm. Pearson correlation analyses were performed to examine the associations between changes from baseline to week 24 in each estimated fluid volume parameter and the clinical variables, including log-transformed NT-proBNP concentration.

All statistical analyses were performed using SAS software version 9.4 (SAS Institute, Cary, NC, USA). A two-sided significance level of P < 0.05 was used for all assessments, and no adjustment for multiplicity was applied in the analyses.

## Results

Among 117 patients randomized, 12 were excluded from analysis owing to loss during follow-up or serious protocol deviation. Accordingly, 105 patients were primarily analyzed (empagliflozin n = 52, placebo n = 53). Detailed baseline characteristics have been reported [16, 17], and were balanced between the treatment groups (Table 1). Briefly, the majority of the subjects were male and had a history of multiple cardiovascular risk factors. Overall, the mean duration of diabetes was 13.3 ± 11.1 years, with a mean glycohemoglobin of 55 ± 9 mmol/mol (7.2 ± 0.8%). A total of 42 patients (40.0%) had a history of HF, and 18 patients (17.1%) had been receiving conventional diuretics at baseline.

**Table 1:** Baseline characteristics of the included patients

	Empagliflozin (n = 52)	Placebo (n = 53)
Age, yrs	65.4 ± 11.1	64.1 ± 9.9
Males	36 (69.2)	36 (67.9)
Systolic blood pressure, mm Hg	132.8 ± 15.2	133.0 ± 14.5
Diastolic blood pressure, mm Hg	76.4 ± 11.5	74.9 ± 9.5
Body mass index, kg/m^2^	26.2 ± 5.1	26. 9 ± 5.5
Hemoglobin, g/dL	14.0 ± 1.6	13.7 ± 1.5
Hematocrit, %	41.6 ± 4.6	41.3 ± 4.2
Uric acid, mg/dL	5.7 ± 1.4	5.3 ± 1.1
Diabetes duration, yrs	13.6 ± 13.2	13.0 ± 8.3
Fasting plasma glucose, mg/dL	141.4 ± 25.0	146.4 ± 34.8
Glycohemoglobin, % (mmol/mol)	7.2 ± 0.8 (55 ± 9)	7.2 ± 0.9 (55 ± 10)
eGFR, mL/min/1.73m^2^	67.0 ± 12.5	69.2 ± 13.9
UACR, mg/g·Cre	32.0 (8.0 to 65.0)	15.3 (7.5 to 41.5)
NT-proBNP, pg/mL	63.0 (31.0 to 180.0)	80.5 (20.0 to 122.0)
High-sensitivity troponin I, pg/mL	3.2 (2.3 to 6.3)	4.1 (2.2 to 8.1)
Past medical history		
Hypertension	41 (78.8)	36 (67.9)
Dyslipidemia	39 (75.0)	38 (71.7)
Heart failure	23 (44.2)	19 (35.8)
Myocardial infarction	12 (23.1)	13 (24.5)
Treatment		
Metformin	25 (48.1)	28 (52.8)
Thiazolidinedione	12 (23.1)	13 (24.5)
DPP-4 inhibitor	37 (71.2)	36 (67.9)
ACE inhibitor or ARB	31 (59.6)	38 (71.7)
Beta-blocker	19 (36.5)	19 (35.8)
MRA	9 (17.3)	5 (9.4)
Diuretic	8 (15.4)	10 (18.9)

Data are expressed as n (%), mean ± SD or median (interquartile)

*ACE* angiotensin-converting enzyme, *ARB* angiotensin receptor blocker, *DPP-4* dipeptidyl peptidase-4, *eGFR* estimated glomerular filtration rate, *MRA* mineralocorticoid receptor antagonist, *NT-proBNP* N-terminal pro-brain natriuretic peptide, *UACR* urinary albumin-creatinine ratio

**Fig. 1:**
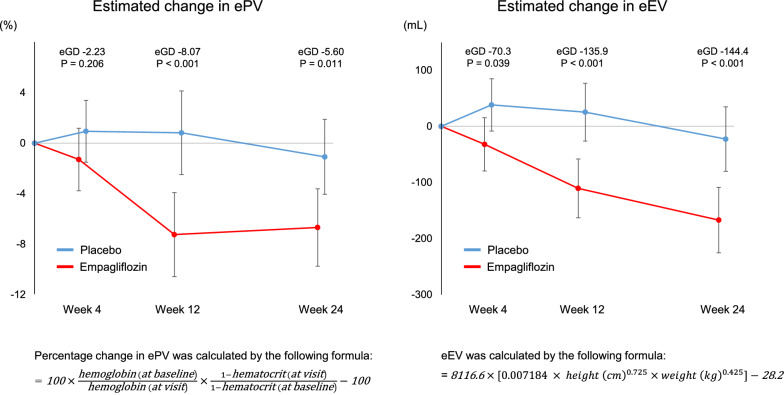
Effect of empagliflozin on estimated fluid volume status through week 24. *eEV* estimated extracellular volume, *eGD* estimated group difference, *ePV* estimated plasma volume

The baseline values and changes in ePV and eEV from baseline to weeks 4, 12, and 24 are shown in Table 2. The baseline values of ePV and eEV were similar in the treatment groups. Empagliflozin reduced ePV and eEV compared to placebo from baseline to week 4 (ePV, non-adjusted mean group-difference − 1.98% [95% CI − 5.54 to 1.57]; eEV − 57.2 mL [95% CI − 124.8 to 10.4]), to week 12 (ePV, − 7.85% [95% CI − 12.65 to − 3.05]; eEV − 143.4 mL [95% CI − 206.5 to − 80.3]), and to week 24 (ePV, − 5.53% [95% CI − 9.84 to − 1.22]; eEV − 145.5 mL [95% CI − 228.4 to − 62.7]). In the adjusted analysis using the MMRM model, relative to placebo empagliflozin significantly reduced ePV by − 8.07% (95% CI − 12.76 to − 3.37) at week 12 and by − 5.60% (95% CI − 9.87 to − 1.32) at week 24; eEV by − 70.3 mL (95% CI − 136.8 to − 3.8) at week 4, − 135.9 mL (95% CI − 209.6 to − 62.3) at week 12, and by − 144.4 mL (95% CI − 226.3 to − 62.4) at week 24 (Fig. 1).

**Table 2:** Changes in estimated fluid volume status at weeks 4, 12, and 24

	Empagliflozin		Placebo		Group difference (95% CI)
ePV	(n)		(n)		
Baseline^a^	51	2472 ± 382	53	2535 ± 432	− 63 (− 222 to 96)
Change from baseline to 4 weeks, %	44	− 1.19 ± 7.06	45	0.79 ± 9.59	− 1.98 (− 5.54 to 1.57)
Change from baseline to 12 weeks, %	46	− 7.42 ± 9.50	46	0.43 ± 13.35	− 7.85 (− 12.65 to − 3.05)
Change from baseline to 24 weeks, %	48	− 6.66 ± 12.57	52	− 1.13 ± 8.98	− 5.53 (− 9.84 to − 1.22)
eEV					
Baseline, mL	52	13,860 ± 1764	53	14,089 ± 1564	− 228 (− 873 to 416)
Change from baseline to 4 weeks, mL	45	− 32.4 ± 172.5	47	24.9 ± 153.6	− 57.2 (− 124.8 to 10.4)
Change from baseline to 12 weeks, mL	46	− 131.2 ± 139.7	48	12.3 ± 166.5	− 143.4 (− 206.5 to − 80.3)
Change from baseline to 24 weeks, mL	50	− 168.3 ± 202.2	52	− 22.8 ± 218.8	− 145.5 (− 228.4 to − 62.7)

Data are expressed as mean ± SD

*CI* confidence interval, *eEV* estimated extracellular volume, *ePV* estimated plasma volume

^a^ePV at baseline was calculated by the Kaplan-Hakim formula

**Fig. 2:**
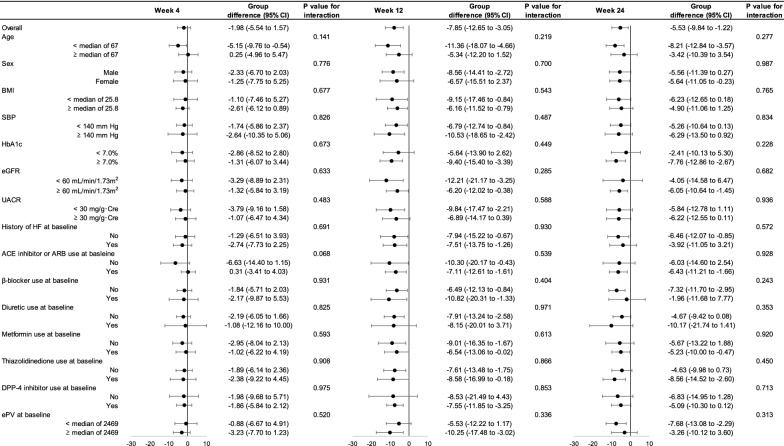
Subgroup analyses of change in ePV through week 24. *ACE* angiotensin-converting enzyme, *ARB* angiotensin receptor blocker, *BMI* body mass index, *CI* confidence interval, *DPP-4* dipeptidyl peptidase-4, *eEV* estimated extracellular volume, *eGFR* estimated glomerular filtration rate, *ePV* estimated plasma volume, *HF* heart failure, *SBP* systolic blood pressure, *UACR* urinary albumin-creatinine ratio

**Fig. 3:**
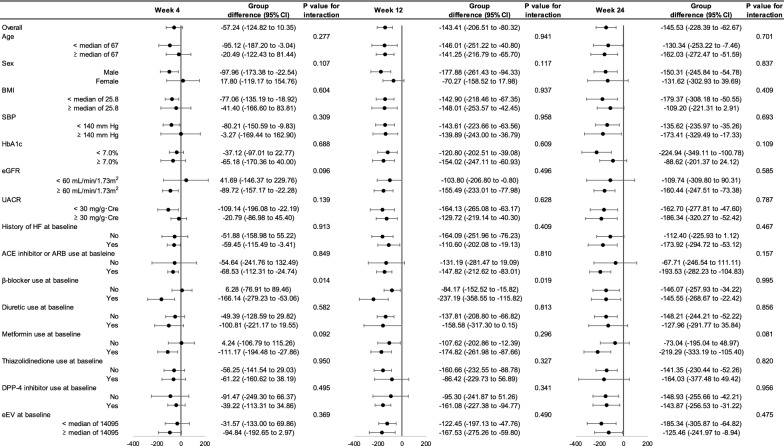
Subgroup analyses of change in eEV through week 24. *eEV* estimated extracellular volume. Others see Fig. 2

The effects of empagliflozin on ePV (Fig. 2) and eEV (Fig. 3) in the total subjects were almost consistent across the different patient subgroups at weeks 4, 12 and 24. Specifically, relative to placebo, empagliflozin reduced those parameters at weeks 12 and 24, regardless of BMI, history of HF, diuretic use, and the corresponding estimated fluid volume at baseline. All P values for interaction, except according to β-blocker use at baseline for eEV at weeks 4 and 12, were > 0.05.

**Fig. 4:**
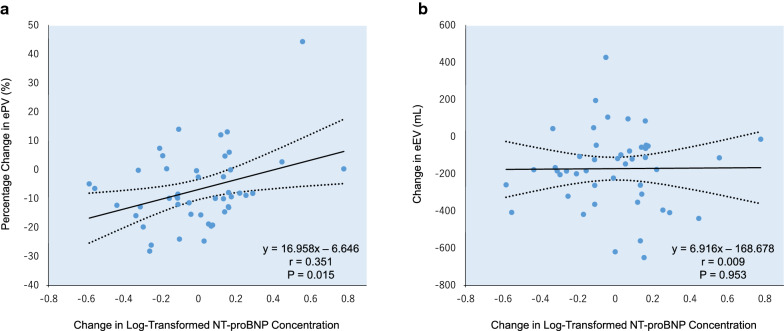
Correlation between changes in estimated fluid volumes and NT-proBNP concentration. **a** Scatterplot detailing the correlation between percentage change from baseline to week 24 in ePV and corresponding change in log-transformed NT-proBNP concentration in the empagliflozin treatment group. **b** Scatterplot detailing the correlation between change from baseline to week 24 in eEV and corresponding change in log-transformed NT-proBNP concentration in the empagliflozin treatment group. The mean regression line (solid line) and 95% confidence intervals (broken line) are displayed. *NT-proBNP* N-terminal pro-brain natriuretic peptide. Others see Fig. 2

Twenty-four weeks of empagliflozin treatment did not change the NT-proBNP concentration (geometric mean 71.3 pg/mL [95% CI 48.7 to 104.4] at baseline and 66.1 pg/mL [95% CI 46.3 to 94.2] at week 24, proportional change 0.995 [95% CI 0.833 to 1.189]). In the continuous analyses, the change from baseline to week 24 in log-transformed NT-proBNP concentration was positively correlated with the corresponding change in ePV, but not with that in eEV (Fig. 4).

Detailed changes in clinical parameters, including body weight, hematocrit, and hemoglobin, from baseline to weeks 4, 12, and 24 are shown in Additional file 1, while changes in other clinical data from baseline to week 24 have been reported previously [16, 17]. There were weak correlations between changes from baseline to week 24 in ePV and glycohemoglobin and estimated glomerular filtration rate, and the change in eEV was correlated with changes in systolic blood pressure and BMI (Table 3). No significant correlations between changes from baseline to week 24 in either of the estimated fluid volumes (ePV and eEV) and other measured parameters were observed.

**Table 3:** Pearson correlations between changes from baseline to week 24 in each estimated fluid volume and clinical variables of interest

Variables	ePV		eEV	
	Coefficient	P value	Coefficient	P value
Systolic blood pressure	0.090	0.542	0.374	0.007
Diastolic blood pressure	0.095	0.520	0.186	0.195
Body mass index	− 0.143	0.333	0.986	< 0.001
Uric acid	− 0.091	0.546	0.132	0.380
Fasting plasma glucose	0.138	0.361	− 0.081	0.591
Glycohemoglobin	− 0.287	0.048	− 0.027	0.855
eGFR^a^	0.407	0.005	− 0.091	0.538
UACR^a^	− 0.011	0.949	0.038	0.817
High-sensitivity troponin I^a^	0.177	0.240	0.252	0.091
Total ketone bodies^a^	0.029	0.855	− 0.013	0.937
RHI^a^	− 0.184	0.215	0.155	0.298

*RHI* reactive hyperemia index. Others see Table 1

^a^Log-transformed

## Discussion

In this *post-hoc* analysis of the EMBLEM trial, the magnitudes of the reductions of the estimated fluid volumes from baseline to 4, 12, and 24 weeks after initiation of empagliflozin 10 mg once daily, relative to placebo, were robust in patients with type 2 diabetes and CVD. This effect was mostly consistent across patient medical backgrounds. Our findings suggest that an early and sustained effect of empagliflozin on volume-related markers is a key mechanism underlying the clinical benefit of the drug.

SGLT2 inhibitors possesses pleiotropic hemodynamic and metabolic actions beyond their glucose-lowering action, causing direct and indirect effects for cardiovascular and renal protection [7]. However, the CVOTs were not primarily designed to determine the mechanisms that might mediate these prognostic relationships, and the mechanisms underlying the clinical benefits of SGLT2 inhibitors remain uncertain. Such benefits are unlikely to be associated with their glucose-lowering action or the diabetic status of patients [20–22]. Interestingly, two recent mediation analyses of data obtained from CVOTs (the EMPA-REG OUTCOME trial and the CANVAS program) suggested that changes in markers of erythropoiesis and volume status were the most important mediators of the decreases in the risk of HHF and cardiovascular death [12, 13]. Thus, this approach revealed possible determinants of the probability of clinical benefit during treatment with SGLT2 inhibitors, and brought much attention to such secondary effects of the drugs.

SGLT2 inhibitors are known to promote osmotic/natriuretic diuresis and to reduce excess fluid volumes without affecting effective intravascular circulating volume, presumably underlying the risk reduction in HHF [9–11]. To estimate changes in fluid volume status during SGLT2 inhibitor therapy, previous studies examined the effects of dapagliflozin or canagliflozin on Strauss formula-based ePV, an established prognostic marker in patients with HF [19, 23]. These analyses showed consistent decreases in a broad range of patients with type 2 diabetes, irrespective of their HF status [14, 24]. More recently, 12 weeks of empagliflozin reduced both ePV and eEV, estimated by the body surface area, in patients with HF and reduced ejection fraction, regardless of diabetic status [15]. In the present analysis, we also examined changes in ePV and eEV following initiation of empagliflozin treatment through 24 weeks in order to extend previous findings to patients with type 2 diabetes at high risk of CVD, specifically similar to the previous CVOTs with SGLT2 inhibitors, and to explore mechanisms underlying the early benefit of SGLT2 inhibitors in that patient population.

In the initial results of the EMBLEM trial, 24 weeks of empagliflozin did not affect endothelial function as assessed by RHI in patients with type 2 diabetes and CVD [16, 17], suggesting that improvement of vascular function was unlikely to have been responsible for the early clinical benefits observed in the CVOTs with SGLT2 inhibitors. In contrast, the present analysis demonstrated reductions of the estimated fluid volumes at week 4 following initiation of empagliflozin that were maintained until 24 weeks. Notably, in the EMPA-REG OUTCOME trial, the risk reduction in HHF was apparent immediately after initiation of empagliflozin, and was also consistent across subgroups stratified by a variety of clinical backgrounds [25]. These findings suggest that the early hemodynamic action of SGLT2 inhibitors and resulting regulation of fluid volume are major factors that contribute to the beneficial impact on cardiovascular events in patients with type 2 diabetes and a high risk of CVD, especially for those with HHF [26, 27].

In the present analysis, a reduction in ePV relative to placebo was apparent at week 12 following initiation of empagliflozin. This is likely due to the previous observation that the erythropoiesis and relevant variables (hematocrit and hemoglobin) that were incorporated into the Strauss formula gradually increase after 4 weeks of SGLT2 inhibitor treatment, and continue over time [28–30]. On the other hand, the effect on eEV, which was based on body surface area, developed soon after initiation of empagliflozin and was also sustained until 24 weeks, regardless of patient medical backgrounds. This finding would be consistent with the sustained body weight loss that has been reported after initiation of SGLT2 inhibitors [28], although the diuretic effect and loss of extracellular volume resulting from SGLT2 inhibition appears to be transient [31, 32]. Therefore, the variations in estimated fluid volumes reflected changes in relevant clinical variables following initiation of empagliflozin treatment.

Regarding the effects of SGLT2 inhibition on erythropoiesis, in 2019 Sano et al. [33] proposed a possible mechanism to explain the increase in hematocrit associated with SGLT2 inhibition. This involved an improvement in the hypoxic microenvironment of the renal tubular interstitium, with SGLT2 inhibition promoting phenotypic reverse-transformation of non-functional myofibroblasts to erythropoietin-producing fibroblasts, thereby stimulating erythropoietin production and subsequent erythropoiesis [33]. Recently, Mazer et al. [34] reported that SGLT2 inhibition with empagliflozin in patients with T2D and coronary artery disease increased early erythropoietin levels. These authors also proposed several SGLT2-associated renal mechanisms that included increased β-hydroxybutyrate levels and expression of hypoxia-inducible factors (HIFs) [34]. Furthermore, the increase in erythropoiesis may be explained by SGLT2 inhibitor-mediated activation of sirtuin-1 signaling and subsequent regulation of imbalances in HIF-1α and HIF-2α [35]. Taken together these findings indicate that increased erythropoiesis associated with SGLT2 inhibition appears to reflect favorable intra-renal responses, rather than simple hemoconcentration through loss of intravascular fluid volume. This possibility suggests that this action, itself, is likely to be the key mediator of the cardiorenal protective effect of SGLT2 inhibitors.

The present study also showed that change in the log-transformed NT-proBNP concentration was weakly but significantly correlated with change in ePV, while no correlation with eEV was observed. Several studies in patients who received HF treatment have also demonstrated that changes in ePV were correlated with those in natriuretic peptide concentration [36]. Although the reason for the difference observed between ePV and eEV is unclear, ePV is a more suitable marker of left ventricular wall stress status, suggesting an easy-to-obtain indicator of cardiac workload. Given the consistent reduction of ePV after initiation of SGLT2 inhibitors, these findings also offer an explanation for the cardiovascular benefits seen in the CVOTs with SGLT2 inhibitors.

Limitations of the present study include that this was a *post-hoc* analysis; that a rather small number of patients were recruited; and that the estimations of fluid volumes were limited to 4, 12, and 24 weeks after the start of treatment. Given the number of analyses performed our data cannot exclude susceptibility to multiplicity and the presence of type I statistical errors. Further studies and large-scale observations are therefore warranted to investigate whether SGLT2 inhibitor-induced reduction in fluid volumes is predictive of long-term clinical benefit in patients with variable clinical characteristics. Most importantly, no direct measures of fluid volume were made in the present study. Both ePV and eEV were influenced by the changes in the variables incorporated in their formulas, although several studies have demonstrated reasonable correlations between estimated and measured (actual) fluid volumes [14, 36]. In particular, given the transient diuretic effect of SGLT2 inhibitor described above, it is possible that estimation of plasma volume status using the Strauss formula might be substantially influenced substantially by SGLT2 inhibitor-induced erythropoiesis. We are therefore unable to exclude the possibility that the present estimated changes in volume status were partially the result of actual changes in plasma volume status. It is necessary to measure SGLT2 inhibitor-induced changes in body composition and fluid status directly using the bioimpedance method [32, 37].

In conclusion, our data demonstrate that empagliflozin substantially reduced the estimated fluid volume parameters in patients with type 2 diabetes and CVD, and that this effect was maintained for 24 weeks. Given the early beneficial effect of empagliflozin on cardiovascular outcomes seen in similar patient populations, our findings suggest an important insight into key mechanisms underlying the clinical benefit of the drug.

## Supplementary Information

**Additional file 1.** Changes in clinical parameters at weeks 4, 12, and 24.

## Data Availability

The datasets analyzed during the current study are available from the corresponding author on reasonable request (tanakaa2@cc.saga-u.ac.jp).

## Abbreviations

BMI: Body mass index
CI: Confidence interval
CVD: Cardiovascular disease
CVOT: Cardiovascular outcome trial
eEV: Estimated extracellular volume
ePV: Estimated plasma volume
HF: Heart failure
HHF: Hospitalization for heart failure
HIF: Hypoxia-inducible factor
MMRM: Mixed-effects models for repeated measures
NT-proBNP: N-terminal pro-brain natriuretic peptide
RHI: Reactive hyperemia index
SGLT2: Sodium glucose co-transporter 2
