# Supplementary material for: Reduction of estimated fluid volumes following initiation of empagliflozin in patients with type 2 diabetes and cardiovascular disease: a secondary analysis of the placebo-controlled, randomized EMBLEM trial
Source: Cardiovasc Diabetol. 2021 Jun 28;20:105. doi: 10.1186/s12933-021-01295-6 (PMC8237440; doi:10.1186/s12933-021-01295-6)
Supplement: Supplementary file 1 — Additional file 1. Changes in clinical parameters at weeks 4, 12, and 24. [file 12933_2021_1295_MOESM1_ESM.docx]

**Additional File 1. Changes in clinical parameters at weeks 4, 12, and 24**

| **Variables** | **Empagliflozin** | | **Placebo** | | **Group difference (95% CI)** |
| --- | --- | --- | --- | --- | --- |
|  | **n** | **Mean ± SD** | **n** | **Mean ± SD** |  |
| Body weight, kg |  |  |  |  |  |
| Baseline | 52 | 68.29 ± 16.59 | 53 | 70.14 ± 15.24 | -1.85 (-8.02 to 4.32) |
| Week 4 | 45 | 66.86 ± 17.15 | 47 | 70.83 ± 15.33 | -3.97 (-10.72 to 2.78) |
| Week 12 | 46 | 66.12 ± 16.76 | 48 | 70.71 ± 15.70 | -4.59 (-11.25 to 2.07) |
| Week 24 | 50 | 66.84 ± 15.98 | 52 | 69.92 ± 14.99 | -3.08 (-9.17 to 3.02) |
| Change from baseline to week 4 | 45 | -0.36 ± 2.04 | 47 | 0.30 ± 1.89 | -0.66 (-1.47 to 0.16) |
| Change from baseline to week 12 | 46 | -1.49 ± 1.62 | 48 | 0.16 ± 2.03 | -1.64 (-2.40 to -0.89) |
| Change from baseline to week 24 | 50 | -1.95 ± 2.45 | 52 | -0.30 ± 2.46 | -1.65 (-2.62 to -0.69) |
| Body mass index, kg/m^2^ |  |  |  |  |  |
| Baseline | 52 | 26.26 ± 5.09 | 53 | 26.80 ± 5.51 | -0.53 (-2.58 to 1.52) |
| Week 4 | 45 | 26.04 ± 5.46 | 47 | 26.98 ± 5.55 | -0.95 (-3.23 to 1.33) |
| Week 12 | 46 | 25.49 ± 5.21 | 48 | 26.93 ± 5.58 | -1.43 (-3.64 to 0.78) |
| Week 24 | 50 | 25.68 ± 4.94 | 52 | 26.71 ± 5.45 | -1.03 (-3.07 to 1.01) |
| Change from baseline to week 4 | 45 | -0.11 ± 0.98 | 47 | 0.12 ± 0.69 | -0.22 (-0.58 to 0.13) |
| Change from baseline to week 12 | 46 | -0.58 ± 0.61 | 48 | 0.06 ± 0.75 | -0.63 (-0.91 to -0.35) |
| Change from baseline to week 24 | 50 | -0.74 ± 0.97 | 52 | -0.10 ± 0.92 | -0.64 (-1.01 to -0.26) |
| Systolic blood pressure, mm Hg |  |  |  |  |  |
| Baseline | 52 | 132.8 ± 15.2 | 53 | 133.0 ± 14.5 | -0.2 (-6.0 to 5.5) |
| Week 4 | 52 | 124.8 ± 14.2 | 53 | 127.9 ± 12.0 | -3.1 (-8.2 to 2.0) |
| Week 12 | 52 | 127.7 ± 13.7 | 53 | 129.0 ± 12.2 | -1.3 (-6.4 to 3.7) |
| Week 24 | 50 | 124.9 ± 14.4 | 52 | 130.6 ± 13.5 | -5.7 (-11.2 to -0.2) |
| Change from baseline to week 4 | 52 | -8.0 ± 12.6 | 53 | -5.1 ± 14.1 | -2.9 (-8.1 to 2.3) |
| Change from baseline to week 12 | 52 | -5.1 ± 15.1 | 53 | -4.0 ± 14.3 | -1.1 (-6.8 to 4.6) |
| Change from baseline to week 24 | 50 | -7.6 ± 16.5 | 52 | -2.1 ± 12.1 | -5.4 (-11.1 to 0.3) |
| Diastolic blood pressure, mm Hg |  |  |  |  |  |
| Baseline | 52 | 76.4 ± 11.5 | 53 | 74.9 ± 9.5 | 1.4 (-2.7 to 5.5) |
| Week 4 | 52 | 72.8 ± 11.4 | 53 | 71.7 ± 9.0 | 1.1 (-2.9 to 5.1) |
| Week 12 | 52 | 72.0 ± 10.2 | 53 | 73.8 ± 9.9 | -1.8 (-5.8 to 2.1) |
| Week 24 | 50 | 72.6 ± 9.2 | 52 | 74.7 ± 11.3 | -2.1 (-6.1 to 2.0) |
| Change from baseline to week 4 | 52 | -3.6 ± 9.9 | 53 | -3.2 ± 9.3 | -0.4 (-4.1 to 3.4) |
| Change from baseline to week 12 | 52 | -4.4 ± 11.0 | 53 | -1.1 ± 8.9 | -3.3 (-7.2 to 0.6) |
| Change from baseline to week 24 | 50 | -3.7 ± 8.7 | 52 | -0.2 ± 9.9 | -3.5 (-7.2 to 0.1) |
| Heart rate, bpm |  |  |  |  |  |
| Baseline | 52 | 73.8 ± 13.3 | 53 | 71.9 ± 9.8 | 1.9 (-2.6 to 6.5) |
| Week 4 | 43 | 78.4 ± 13.8 | 46 | 73.1 ± 10.5 | 5.3 (0.1 to 10.5) |
| Week 12 | 46 | 74.6 ± 13.3 | 47 | 74.0 ± 11.3 | 0.6 (-4.5 to 5.7) |
| Week 24 | 50 | 74.2 ± 16.1 | 51 | 70.9 ± 10.2 | 3.3 (-2.1 to 8.6) |
| Change from baseline to week 4 | 43 | 2.6 ± 9.7 | 46 | 0.6 ± 6.3 | 2.0 (-1.4 to 5.5) |
| Change from baseline to week 12 | 46 | 0.7 ± 12.8 | 47 | 1.6 ± 8.6 | -0.9 (-5.4 to 3.7) |
| Change from baseline to week 24 | 50 | 0.5 ± 15.4 | 51 | -0.6 ± 8.4 | 1.2 (-3.8 to 6.1) |
| Fasting plasma glucose, mg/dL |  |  |  |  |  |
| Baseline | 50 | 141.4 ± 25.0 | 52 | 146.4 ± 34.8 | -5.0 (-16.9 to 6.9) |
| Week 4 | 42 | 130.5 ± 28.6 | 46 | 142.5 ± 31.1 | -12.0 (-24.6 to 0.7) |
| Week 12 | 45 | 129.1± 29.0 | 46 | 149.8 ± 36.8 | -20.6 (-34.4 to -6.8) |
| Week 24 | 47 | 127.8± 25.3 | 51 | 145.5 ± 42.7 | -17.7 (-31.7 to -3.8) |
| Change from baseline to week 4 | 41 | -9.0 ± 27.1 | 45 | -3.0 ± 31.3 | -6.0 (-18.6 to 6.5) |
| Change from baseline to week 12 | 44 | -8.9 ± 26.6 | 45 | 4.5 ± 40.3 | -13.4 (-27.8 to 1.0) |
| Change from baseline to week 24 | 46 | -17.9 ± 22.0 | 51 | -0.8 ± 37.6 | -17.1 (-29.4 to -4.8) |
| Glycohemoglobin, % |  |  |  |  |  |
| Baseline | 52 | 7.15 ± 0.82 | 53 | 7.20 ± 0.88 | -0.04 (-0.37 to 0.29) |
| Week 4 | 51 | 6.95 ± 0.69 | 53 | 7.17 ± 0.88 | -0.22 (-0.53 to 0.08) |
| Week 12 | 52 | 6.79 ± 0.70 | 52 | 7.20 ± 0.86 | -0.41 (-0.71 to -0.10) |
| Week 24 | 48 | 6.91 ± 0.64 | 52 | 7.26 ± 0.86 | -0.35 (-0.65 to -0.05) |
| Change from baseline to week 4 | 51 | -0.21 ± 0.31 | 53 | -0.02 ± 0.31 | -0.19 (-0.31 to -0.07) |
| Change from baseline to week 12 | 52 | -0.36 ± 0.46 | 52 | 0.01 ± 0.64 | -0.37 (-0.58 to -0.15) |
| Change from baseline to week 24 | 48 | -0.25 ± 0.49 | 52 | 0.07 ± 0.71 | -0.32 (-0.56 to -0.07) |
| Hematocrit, % |  |  |  |  |  |
| Baseline | 51 | 41.58 ± 4.56 | 53 | 41.33 ± 4.15 | 0.25 (-1.45 to 1.95) |
| Week 4 | 44 | 42.00 ± 4.48 | 45 | 41.31 ± 3.91 | 0.69 (-1.08 to 2.47) |
| Week 12 | 46 | 43.74 ± 4.73 | 46 | 41.19 ± 4.57 | 2.55 (0.62 to 4.47) |
| Week 24 | 49 | 43.58 ± 4.80 | 52 | 41.72 ± 3.75 | 1.86 (0.15 to 3.57) |
| Change from baseline to week 4 | 44 | 0.56 ± 1.86 | 45 | -0.08 ± 2.30 | 0.64 (-0.24 to 1.52) |
| Change from baseline to week 12 | 46 | 2.17 ± 2.67 | 46 | 0.07 ± 3.44 | 2.11 (0.83 to 3.39) |
| Change from baseline to week 24 | 48 | 2.05 ± 3.59 | 52 | 0.38 ± 2.45 | 1.68 (0.44 to 2.91) |
| Hemoglobin, g/dL |  |  |  |  |  |
| Baseline | 51 | 13.96 ± 1.59 | 53 | 13.73 ± 1.46 | 0.23 (-0.36 to 0.82) |
| Week 4 | 44 | 13.99 ± 1.53 | 45 | 13.69 ± 1.45 | 0.30 (-0.33 to 0.93) |
| Week 12 | 46 | 14.58 ± 1.55 | 46 | 13.69 ± 1.54 | 0.89 (0.25 to 1.53) |
| Week 24 | 49 | 14.54 ± 1.60 | 52 | 13.83 ± 1.34 | 0.71 (0.12 to 1.29) |
| Change from baseline to week 4 | 44 | 0.06 ± 0.56 | 45 | -0.02 ± 0.76 | 0.09 (-0.20 to 0.37) |
| Change from baseline to week 12 | 46 | 0.62 ± 0.81 | 46 | 0.05 ± 1.06 | 0.56 (0.17 to 0.95) |
| Change from baseline to week 24 | 48 | 0.58 ± 0.98 | 52 | 0.12 ± 0.71 | 0.46 (0.12 to 0.80) |

CI = confidence interval; SD = standard deviation.
